# Intrathymic Localization of Melanoma: A Brief Report of Two Cases and a Review of the Literature

**DOI:** 10.3390/diagnostics13122017

**Published:** 2023-06-09

**Authors:** Giorgio Cannone, Vincenzo Verzeletti, Francesco Fortarezza, Federica Pezzuto, Roberta Polverosi, Eleonora Faccioli, Giovanni Maria Comacchio, Andrea Dell’Amore, Federico Rea, Marco Schiavon, Fiorella Calabrese

**Affiliations:** 1 Thoracic Surgery Unit, Department of Cardiac, Thoracic, Vascular Sciences and Public Health, University of Padua, 35122 Padua, Italy; 2 Pathology Unit, Policlinic University of Padua, 35131 Padua, Italy; 3 Pathology Unit, Department of Cardiac, Thoracic, Vascular Sciences and Public Health, University of Padua, 35122 Padua, Italy; 4 Antoniano Diagnostic Institute, 35123 Padua, Italy

**Keywords:** melanoma, thymus, metastases, BRAF

## Abstract

Intrathymic localizations of melanoma represent a very rare entity, with fewer than ten cases of intrathymic melanoma described in the literature. Herein, we describe two cases of patients who underwent surgical removal of a thymic mass at our thoracic surgery department between 2015 and 2022. The final pathological examination revealed a malignant melanoma in both cases; we therefore carried out a literature review to identify such rare and similar cases. In the first case, the intrathymic localization of melanoma was the first manifestation of the disease, posing a dilemma regarding the metastatic and primitive nature of the neoplasm. The second case described a thymic metastasis from a known previous cutaneous melanoma, for which the patient had successfully been treated six years earlier. After carefully reviewing the literature, we identified only six cases of verified primary intrathymic melanomas and one case of intrathymic metastasis resulting from melanoma previously described. Pathologists should be aware of the occurrence of this rare entity and mindful of the differential diagnoses. Several tools, including immunostaining of melanocytic markers and molecular investigations, are mandatory for final pathological diagnosis.

## 1. Introduction

Malignant melanoma is a malignancy of melanocytes, which originates from the neural crest formed on the neural tube and usually occurs in the skin. Visceral organs such as the lungs, liver, and brain are commonly involved due to distant metastases from primary melanoma of the skin [1]. There are also extracutaneous sites of primitive melanoma including ocular, mucosal, leptomeningeal, and internal organ melanomas. In the literature, it is debated as to whether the melanomas primarily diagnosed in internal organs (i.e., the larynx, esophagus, lungs, rectum, ovaries, and uterus) are actually primary neoplasms or whether they are metastases from melanoma of unknown origin. In favor of the first theory, it was assumed that the melanocytes from the neural tube of the neural crest can migrate to different parts of the body during the early embryological stages. This could be a possible etiopathogenetic mechanism of internal organ melanoma [2,3]. On the other hand, other studies and evidence suggest that melanomas primarily diagnosed in unusual visceral locations are metastases of melanomas of unknown origin, probably from completely regressed or misdiagnosed skin melanoma [4]. The thymus is an exceptional localization of melanoma, with fewer than ten primary intrathymic melanomas and only one report of intrathymic metastasis described in the literature [5,6,7,8,9,10,11]. We present two cases of thymic localization of melanoma misdiagnosed as a primary thymic tumor by clinicians and that were subjected to surgery.

## 2. Materials and Methods

Between 2015 and 2022 at our thoracic surgery department, we came across two particular cases of patients who had undergone surgical thymectomy, both of which turned out to be malignant melanoma during the final pathological examinations. We consequently carried out a literature review to identify similar cases. Medline-indexed research was performed on the topic, using PubMed as the main database, using the string “Thymus/Thymic Melanoma” below on the 31st of December 2022. Subsequently, each of the authors independently assessed the eligibility of the studies by screening the article titles and abstracts and then decided on the inclusion by reading the full text of the selected works.

## 3. Results

### 3.1. Case 1

**Figure 1:**
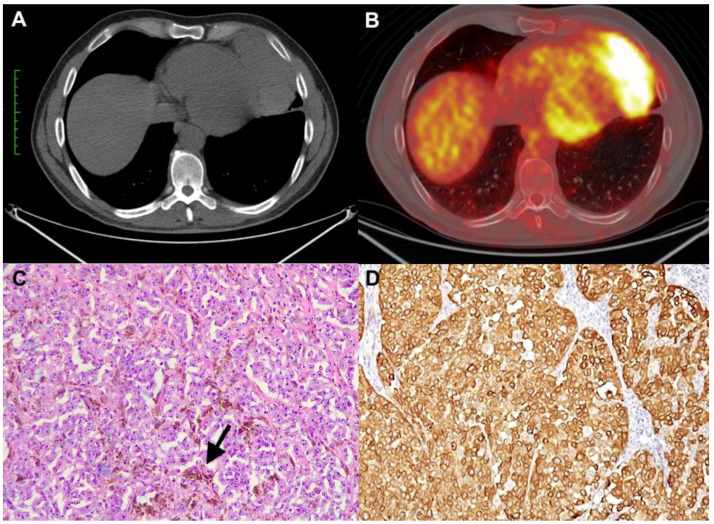
CT scan showing a voluminous mass of the anterior and inferior mediastinum (**A**) with high pathological uptake (SUVmax: 20.8) (**B**). Histologically, the tumor was composed of large and nucleolated cells with deposition of melanic pigment (arrow) (**C**) (hematoxylin and eosin stain, original magnification ×100)). The neoplastic cells were strongly positive for Melan A (**D**) (immunohistochemistry, original magnification ×200)).

A 53-year-old man with an unremarkable past medical history presented to the local emergency room complaining of breathlessness. The chest X-ray showed gross widening of the mediastinum with a well-defined homogenous radio-opaque shadow. A contrast-enhanced chest computerized tomography (CT) scan demonstrated a mediastinal mass with a size of 10.5 cm × 4 cm, strictly adherent to the diaphragm and pericardium (Figure 1A). The diagnostic work-up was concluded with a positron emission tomography (PET) scan that showed pathological uptake only in the mass with a standard uptake volume (SUVmax) of 20.8 (Figure 1B). Because this finding was suggestive of a primary neoplastic process of the anterior mediastinum, likely a thymoma, surgery was planned. Through a left mini-thoracotomy approach, thymectomy en bloc with partial pericardium resection and an atypical resection of the left lower lobe were performed. The procedure was uneventful, and the patient was discharged on the fifth postoperative day. The gross specimen revealed a solid encapsulated mass with a maximum diameter of 10 cm, which was brownish at the cut surface. Histologically, the tumor was composed of atypical epithelioid cells, with large eosinophilic cytoplasm, prominent nucleoli, and a solid growth pattern. Abundant melanic pigment was also present (Figure 1C). There were thymic remnants at the periphery of the tumor. The immunophenotypic panel showed strong positivity for melanocytic differentiation markers: S100, Melan A/MART1 (Figure 1D), HMB45, and SOX10. Immunoreactions for cytokeratin, CD5, CD117, p40, PAX8, and neuroendocrine markers were negative. No mutations of *BRAF* were detected by the real-time PCR method and *EWSR* rearrangement by fluorescence in situ hybridization (FISH). An attempt was made to carry out more in-depth molecular characterization using next-generation sequencing methodology, but the characteristics of the sample (extensive necrotic phenomena) did not allow for adequate DNA quality for analysis. The final diagnosis of malignant melanoma was made. No primary cutaneous and non-cutaneous melanomas were detected upon dermatological, ophthalmological, and gastroenterological examination. After six months, a polypoid endoluminal mass of 4 cm in the distal ileum was identified during radiological follow-up. Surgical resection was performed. The final pathological examination was conclusive for malignant melanoma. Following a multidisciplinary discussion, considering the radicality of the surgical interventions, it was decided not to perform adjuvant treatment, and the patient is under follow-up. Currently, the patient is still alive and free of disease seven years after the first surgery.

### 3.2. Case 2

**Figure 2:**
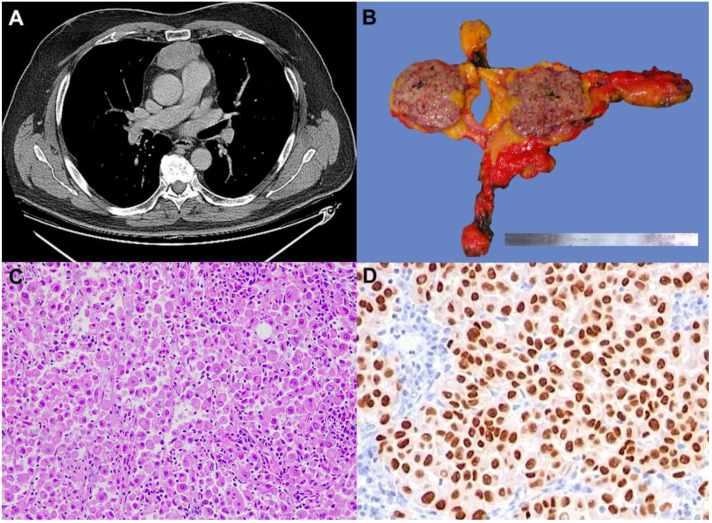
CT scan showing a well-defined retrosternal mediastinal mass (**A**). Gross specimen showing a nodular mass, which is brownish at the cut surface (**B**). The tumor was composed of large and atypical cells with abundant eosinophilic cytoplasm (**C**) (hematoxylin and eosin stain, original magnification ×100). Tumor cells showing nuclear positivity for SOX10 (**D**) (immunohistochemistry, original magnification ×200)).

The second patient was a 62-year-old man with a history of skin melanoma of the cervical region which had been surgically resected in 2016. The pathological stage was pT2a, and the tumor harbored the *BRAF* V600E mutation. One year later a distant relapse to the right foot occurred and was treated with radiotherapy and immunotherapy. After almost five years of controlled disease, a total body CT scan revealed a retrosternal well-defined mediastinal mass of 27 × 30 × 44 mm (Figure 2A), hypermetabolic (SUV = 11.8) at 18-FDG-PET/CT scan. The case was discussed in a multidisciplinary setting; considering the unique localization without metastases in other sites and the radiological features of the mass, a surgical approach was proposed because a primitive neoplasm of the thymus was suspected. A standard robotic-assisted thymectomy was performed. Gross examination showed a brownish nodular mass (Figure 2B). At histology, the tumor was composed of irregularly shaped and eosinophilic neoplastic cells, strongly and diffusely immunoreactive for melanocytic markers (SOX10, Melan A, S100, HMB45). Based on histological and immunohistochemical examination, the diagnosis of intrathymic metastasis of melanoma was made (Figure 2C,D). A large panel of NGS was carried out to identify rare mutations or co-mutations that may have occurred. The tumor showed *BRAF* mutation (V600E) as the primitive melanoma. No additional mutation has been reported. After surgery, the patient started adjuvant treatment with Nivolumab at a dosage of 480 mg every 4 weeks. Currently, the patient has completed eight cycles of treatment with no reported toxicities. One year after surgery, the patient remains under follow-up and is in good health.

## 4. Discussion

Although the thymus is a frequent site of endothoracic neoplasms, non-thymic epithelial tumors and intrathymic metastases are certainly very singular entities [12]. In the literature, reports of intrathymic melanomas are rare, all listed in Table 1. The finding that intrathymic localization is the first manifestation of melanoma raises the dilemma of its origin. In 1965, Misugi et al. described the case of a melanotic progonoma of the posterior mediastinum in a newborn. The authors performed a detailed ultrastructural study that showed the findings of neurogenic cells and non-retinal melanocytes of the tumor, proposing the possibility of a neural crest origin of neoplastic cells [13]. The hypothesis that a melanoma may arise in the thymus is strengthened by the case report by Fushimi et al. [5], where the authors described several foci of pigmented benign nevus cells associated with a thymic melanoma, supporting the theory of a malignant transformation. In the first case of the present report, although primary melanomas were not identified in any of their possible common locations, a totally regressed skin tumor cannot be completely ruled out. Das Gupta et al. demonstrated that primary skin melanomas may spontaneously regress and the only clinical manifestation in affected patients is the presence of metastatic melanoma in the regional lymph nodes or in the visceral organs [14]. Recent molecular evidence, in a series of so-called only-lung melanomas, also shows a high percentage of mutation signatures of ultraviolet radiation, thus supporting the metastatic nature of lung melanoma from regressed skin lesions rather than a true primitive neoplasm [15].

The routine molecular analyses (*BRAF* mutations) may not be relevant in distinguishing metastases from skin melanoma and visceral primitive forms. Indeed, the *BRAF* mutation, a common mutation found in the metastatic setting, can be detected in up 50% of visceral localization as well [16]. A deep molecular approach, possibly also with high-throughput molecular analyses, would be desirable in a series of thymic and, in general, visceral melanomas to further investigate this aspect and properly address the therapeutic approach.

The difficulties in the definition of a primary or metastatic setting leads to uncertainty in the follow-up. Indeed, considering the rarity of the disease, the clinical follow-up requires a multidisciplinary evaluation involving oncologists and dermatologists to achieve more appropriate patient management, as was the case in our cases. Specifically, both of our patients underwent regular chest and abdomen CT scans. As reported in previous studies [17], the choice of performing a PET/CT in the follow-up of melanoma patients could be associated with a high frequency of false positive findings. Thus, we would leave this technique as a second-level strategy, used only if suspicious findings arise.

The problem of the exceptionality of this occurrence remains unsolved due to the lack of studies that can definitively determine the biology and the carcinogenic mechanisms of the tumor. Pathologists should be aware of this rare occurrence to include melanocytic markers in the diagnostic workflow of poorly differentiated thymic neoplasms as well. Moreover, it should be considered that these markers are not entirely specific for determining the origin of the melanoma; they may also be positive for other entities, such as malignant peripheral nerve sheath tumors, PEComas, and clear cell sarcomas. The last one represents a diagnostic challenge, sharing morphological and immunohistochemical similarities with melanoma, thus requiring the confirmation of *EWSR* translocation through molecular/cytogenetic analyses, as was performed in one of our cases. Even more interesting is the description of an intrathymic metastasis of melanoma, which was diagnosed in one of ours and confirmed by clinical-pathological evidence and molecular analyses. To the best of our knowledge, this is the second case described in the literature, with the most peculiar aspect of our case being that, despite the good control of the disease, the thymic tumor progressed independently [15]. This may be explained by the fact that the thymus is an “immunological sanctuary” in which immunotherapy may fail to be effective. A recent study showed that chemotherapy may not be useful against intrathymic melanoma cell clones. Particularly, it was observed that doxorubicin triggers nonmalignant thymic cells, causing thymocyte death and promoting an inflammatory thymic microenvironment. This inflammatory condition causes the thymic-harbored tumor cells to acquire a chemo-resistant state, facilitating the thymus to form a tumor cell reservoir [18]. Thus, in the context of systemic therapy resistance, surgery could play a significant role.

## 5. Conclusions

In exceptional cases, the thymus can be the site of melanoma, either primitive or metastatic. With the present report, we would like to emphasize this occurrence and make pathologists more aware of this rare entity and make them mindful of differential diagnoses.

## Figures and Tables

**Table 1:** Previously published and present cases of intrathymic melanoma.

References	Number of Cases	Primitive or Metastatic	Immunohistochemistry	Outcome
Fushimi et al. [5]	1	Primitive: presence of intrathymic nevus	S100 and HMB45 positive in melanoma cells; S100 positive and HMB45 negative in nevus cells	Lung metastases after 1 year
Shimizu et al. [6]	1	Primitive	HMB45, S100, NES, and Chromogranin positive	Alive with no evidence of disease (54 months)
Taniguchi et al. [8]	1	Primitive	HMB45 and S100 positive	Multiple bone metastases after 5 months, alive with the disease (14 months)
Alli et al. [7]	1	Primitive	HMB45 and S100 positive; weak positivity for cytokeratin and neuroendocrine markers, and CD34 negative	Lung metastases after 14 months
Bavi et al. [9]	1	Primitive	S100, Vimentin, and HMB45 positive; CD5 and cytokeratin negative	Post-operative death
Knipe et al. [10]	1	Metastatic	Melan A, HMB45, and S100 positive	Not available
Katsura et al. [11]	1	Primitive	HMB45 and S100 positive	Free of disease
Present case (1)	1	Primitive	Melan A, HMB45, SOX10, and S100 positive; *BRAF* and *EWSR* wild-type	Free of disease
Present case (2)	1	Metastatic	Melan A, HMB45, SOX10, and S100 positive; *BRAF* V600E mutation	Free of disease

## Data Availability

Data sharing is not applicable to this article.
